# A Tale of Two Cysts: Steatocystoma Multiplex and Eruptive Vellus Hair Cysts—Two Case Reports and a Review of the Literature

**DOI:** 10.1155/2017/3861972

**Published:** 2017-04-05

**Authors:** Rachel J. Waldemer-Streyer, Ellen Jacobsen

**Affiliations:** College of Medicine, University of Illinois-Chicago, Urbana Campus, 506 South Mathews Ave., 190 Medical Sciences Building, MC-714, Urbana, IL 61801, USA

## Abstract

*Background*. Steatocystoma multiplex (SM) and eruptive vellus hair cysts (EVHC) are uncommon benign tumors of the pilosebaceous unit. Both SM and EVHC are characterized by smooth, asymptomatic papules or nodules, most commonly presenting on the chest, limbs, and abdomen. Most cases of SM and EVHC are sporadic, although less common autosomal dominant inherited forms have been reported.* Main Observation*. In this report we present two cases of cutaneous cysts exhibiting characteristics of either SM or EVHC. Both patients presented with numerous 1-2 mm asymptomatic papules and responded well to surgical expression by incision and drainage (I&D).* Conclusion*. SM and EVHC are similar in clinical presentation and management. Previously reported “hybrid-type” tumors present strong evidence for a relationship between the two lesions pathologically. Due to potential similarity of EVHC and SM cyst contents, I&D and subsequent microscopic examination cannot definitely differentiate between EVHC, SM, and hybrid cysts.

## 1. Introduction

Steatocystoma multiplex (SM) and eruptive vellus hair cysts (EVHC) are uncommon benign tumors of the pilosebaceous unit. SM is characterized by multiple smooth asymptomatic papules or nodules, usually presenting on the chest, limbs and axillae, back, or abdomen. Facial involvement is less frequently noted but has been reported [1, 2]. Eruptive vellus hair cysts (EVHC) are a related cutaneous tumor and feature a similar clinical presentation with regard to appearance and spatial distribution, although facial EVHCs are a noted variant [3]. Both SM and EVHC are most likely to emerge during adolescence or early adulthood [1, 3]. Clinical diagnosis of these disorders can be a challenge due to their relative rarity of occurrence. Here we describe two patients with cutaneous cysts exhibiting the features of either SM or EVHC.

## 2. Case Reports

### 2.1. Case Report #1

**Figure 1:**
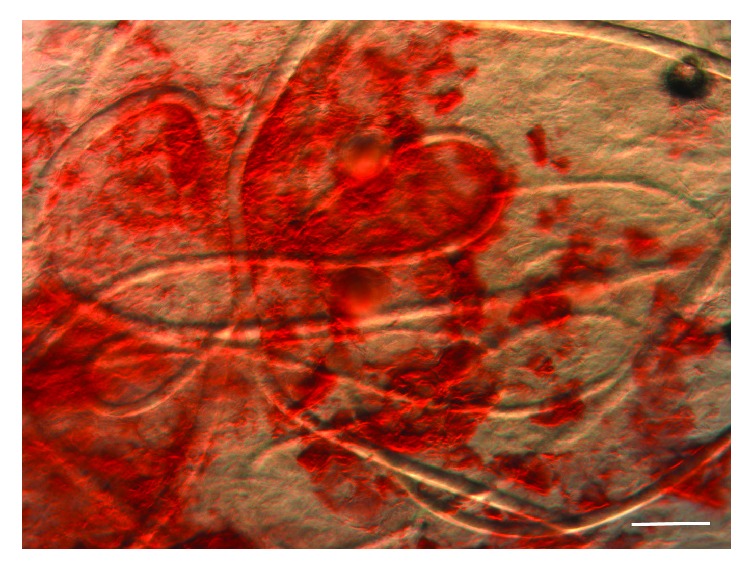
Vellus hairs present in cysts from clinical presentation case #1, a 15-year-old male patient. Cyst contents were expressed via incision and drainage and mounted with a coverslip without KOH. Scale bar = 100 microns.

**Figure 2:**
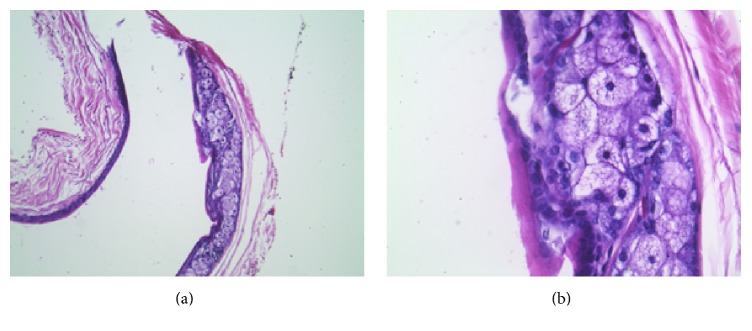
Representative photomicrograph of a steatocystoma stained with hematoxylin and eosin (H&E) at 100x (a) and 400x (b) magnification. Abundant sebaceous input is clearly visible in the cyst wall.

A 15-year-old Caucasian male presented with a six-year history of multiple noninflamed, nonumbilicated papules on his anterior chest and abdomen. The initial eruption in 2009 of white and pink perifollicular 1-2 mm papules was especially numerous (>25) over the sternum, where they developed in a diamond-shaped pattern. No family history of similar dermatological disorders was noted. This eruption was clinically diagnosed as steatocystoma multiplex and showed significant improvement after five years of treatment with a topical 10% glycolic acid lotion. I&D expression of the few remaining lesions resulted in oily, curd-like material containing multiple vellus hairs (Figure 1). While this patient's lesions were not biopsied, a representative photomicrograph of a steatocystoma is shown in Figure 2. Interestingly, the lesions were preceded by a severe case of molluscum contagiosum that spanned the patient's chest, abdomen, back, and buttocks and was treated with 5% imiquimod cream. In addition to molluscum contagiosum, the patient's history was positive for atopic eczema and pityriasis alba on other areas of his body. Concomitant dermatological disorders included acne in the form of open and closed comedones on his face, neck, and chest. The SM lesions were distinct from the acne lesions, the latter of which were erythematous and inflamed.

### 2.2. Case Report #2

**Figure 3:**
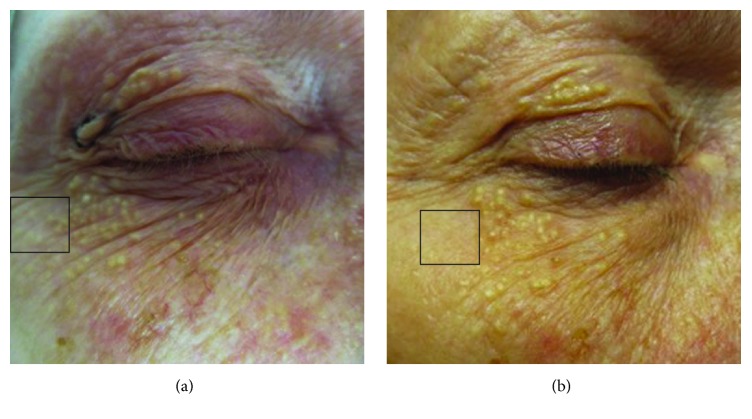
(a) Clinical presentation case #2, a 67-year-old female patient, presurgery. Numerous periorbital 1-2 mm white and yellow papules are shown. Papules inside the highlighted region (black box) were removed surgically. (b) Clinical presentation case #2, one year after surgery. No recurrence of removed papules was observed (boxed region).

**Figure 4:**
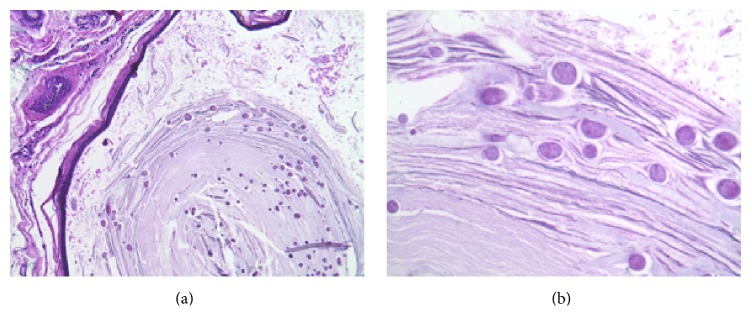
Representative photomicrograph of EVHC stained with H&E at 100x (a) and 400x (b) magnification. Keratin and numerous vellus hair shafts are visible in the cyst lumen.

A 67-year-old Caucasian female presented with numerous 1-2 mm white and yellow papules in a bilateral periorbital distribution (Figure 3(a)), which had emerged over the course of 15 years. Favre-Racouchot syndrome was initially suspected due to her age, cyst location, and concomitant solar elastosis. However, the papules that present with Favre-Racouchot syndrome are often yellow or brown in appearance and accompanied by open comedones, in contrast to our patient. Thus, several of these papules were removed and evaluated pathologically, revealing a mixture of milia and small cysts consistent with the diagnosis of eruptive vellus hair cysts; these contained multiple small lumenal degenerating hair shafts. A representative photomicrograph of an EVHC lesion is shown in Figure 4. Several of this patient's cysts were removed surgically for cosmetic reasons. One year after surgery, no recurrence of the expressed cysts was noted (Figure 3(b)). The patient's history was significant for milia, actinic keratoses, and discoid lupus erythematosus lesions on the scalp.

## 3. Discussion

Many similarities exist between SM and EVHC in terms of clinical presentation. Both SM and EVHC are most likely to emerge during adolescence or early adulthood [1, 3]. However, in their review of EVHC cases, Torchia et al. noted the presence of an interesting subgroup of patients that developed EVHC after age 35. Many of the individuals in this subgroup were women who presented with facial cysts rather than more common sites on the trunk and limbs [3], similar to our second patient. Our female patient also exhibited numerous milia, benign keratinous cysts that commonly present periorbitally. However, current literature does not seem to suggest a general correlation between milia and EVHC formation [3].

Most cases of SM and EVHC are sporadic, although familial cases of each have been reported and are transmitted in an autosomal dominant fashion [1–6]. It is notable that, in our first patient, SM developed shortly after a severe case of molluscum contagiosum and a general history of atopy. As the causes of nonhereditary SM are unknown, it is tempting to speculate that the imiquimod treatment and subsequent robust immune response may have played a role in the development of his cysts.

The true differentiation between SM and EVHC occurs at the histological level. Histological examination of a biopsied SM cyst will reveal thin walls of stratified squamous epithelium without a granular layer, typically with abundant sebaceous input (Figure 2). The cyst is frequently connected to the epidermis by a tissue cord originating from the infundibulum of the pilosebaceous unit [1]. Each steatocystoma is associated with a hair follicle, which can result in retention of trapped vellus hairs in the cystic lumen, as seen in our first patient [1, 2, 7]. Notably, the lesions seen in SM are bona fide tumors, rather than retention cysts caused by blockage of a sebaceous gland [1, 7]. The lining of the cystic cavity typically retains the undulated eosinophilic structure characteristic of the sebaceous duct, from which these hamartomas originate [2].

EVHCs originate from the isthmus or infundibulum of the pilosebaceous unit and thus are often lined by squamous epithelium containing a granular layer, in contrast to SM. These cysts also typically contain laminated keratin and multiple vellus hairs (Figure 4). Due to their different origins in the pilosebaceous unit, SM and EVHC cysts also differ in the classes of keratin found in their walls; while both cysts express keratin 17, only steatocystomas demonstrate expression of keratin 10 [8]. Interestingly, I&D and subsequent microscopic examination of cyst contents have been suggested as a less-invasive alternative to punch biopsy to diagnose EVHC, based on the presence of vellus hairs [9–11]. We disagree with this assertion, as SM lesions can also exhibit vellus hair inclusion, as shown in Figure 1 and reported in other sources [1, 2, 7]. Thus, histologic examination of the biopsied cyst wall and contents remains the gold standard to definitively differentiate between SM and EVHC. However, given the relative ease of examining cyst contents after I&D and the general rule that EVHC lesions are likely to contain a higher number of vellus hairs than those of SM, this procedure may still be useful in a clinical setting.

Intriguingly, there have been various reports of patients presenting with both SM and EVHC concurrently, or with hybrid cysts that exhibit features of both disorders [2, 6, 12–15]. Such reports have prompted speculation that SM and EVHC may be related and on the continuum of one unified disease process [2, 6, 14]. Ohtake et al. were the first to suggest that these hybrid-type tumors may be the result of cystic changes near the junction of the pilosebaceous duct, rather than discrete zones of the hair follicle [15]. Further investigations into the biological mechanisms regulating pilosebaceous tumor development may prove rewarding for our scientific understanding of the relationship between SM and EVHC.

Ultimately, the distinction between SM and EVHC may be primarily an academic one. Both lesions respond to similar treatment modalities and the problems they present are predominantly cosmetic, as they are rarely correlated to any serious genetic syndromes. Noted interventions with a history of efficacy include expression via surgical incision, excision, or needle aspiration; retinoic acid; and Erbium:YAG or CO_2_ lasers [3, 12, 16]. While there have been some reports of spontaneous resolution of EVHC [3], this appears to be a minority of cases.

## 4. Conclusion

Our report details two cases of uncommon pilosebaceous tumors, steatocystoma multiplex, and eruptive vellus hair cysts. Despite clear histological differences between these lesions, SM and EVHC are similar in clinical presentation and management. “Hybrid-type” tumors present strong evidence for a relationship between the SM and EVHC pathologically. Due to their relative rarity of occurrence, familiarity with cyst presentation in these lesions will prove useful for accurate clinical diagnosis.
